# Genomic Analysis of the Pacific Oyster (*Crassostrea gigas*) Reveals Possible Conservation of Vertebrate Sex Determination in a Mollusc

**DOI:** 10.1534/g3.114.013904

**Published:** 2014-09-11

**Authors:** Na Zhang, Fei Xu, Ximing Guo

**Affiliations:** * National & Local Joint Engineering Laboratory of Ecological Mariculture, Institute of Oceanology, Chinese Academy of Sciences, Qingdao, Shandong 266071, China; † Haskin Shellfish Research Laboratory, Institute of Marine and Coastal Sciences, Rutgers University, Port Norris, New Jersey 08349; ‡ University of Chinese Academy of Sciences, Beijing 100049, China

**Keywords:** sex determination, *doublesex*, *Sry*, *FoxL2*, oyster, Mollusca

## Abstract

Despite the prevalence of sex in animal kingdom, we have only limited understanding of how sex is determined and evolved in many taxa. The mollusc Pacific oyster *Crassostrea gigas* exhibits complex modes of sexual reproduction that consists of protandric dioecy, sex change, and occasional hermaphroditism. This complex system is controlled by both environmental and genetic factors through unknown molecular mechanisms. In this study, we investigated genes related to sex-determining pathways in *C. gigas* through transcriptome sequencing and analysis of female and male gonads. Our analysis identified or confirmed novel homologs in the oyster of key sex-determining genes (*SoxH* or *Sry*-like and *FoxL2*) that were thought to be vertebrate-specific. Their expression profile in *C. gigas* is consistent with conserved roles in sex determination, under a proposed model where a novel testis-determining *CgSoxH* may serve as a primary regulator, directly or indirectly interacting with a testis-promoting *CgDsx* and an ovary-promoting *CgFoxL2*. Our findings plus previous results suggest that key vertebrate sex-determining genes such as *Sry* and *FoxL2* may not be inventions of vertebrates. The presence of such genes in a mollusc with expression profiles consistent with expected roles in sex determination suggest that sex determination may be deeply conserved in animals, despite rapid evolution of the regulatory pathways that in *C. gigas* may involve both genetic and environmental factors.

Sexual reproduction is one of the most prevailing and remarkable phenomena in biology. It is found in almost all groups of multicellular animals. The differentiation of two sexes, sometimes with spectacular dimorphism, has long fascinated human curiosity. How sex is determined and evolved remains a major question in modern biology. Why sex is necessary at all given the high cost of producing males has not been adequately answered (Hartfield and Keightley 2012). Assuming sexual production provides critical benefits, it is intriguing that sex is not always genetically determined to ensure a 1:1 sex ratio. In many organisms, sex may be determined by environmental factors or modified by hermaphroditism or sex change (Guo *et al.* 1998; Devlin and Nagahama 2002; Merchant-Larios and Diaz-Hernandez 2013). These variations could be adaptations for reducing the cost of sex while preserving meiosis, but testing this and other hypotheses on the evolution of sex requires a good understanding of molecular mechanisms of sex determination in diverse taxa.

Studies in model organisms have revealed key genes and complex pathways involved in sex determination and differentiation. In mammals, male determination begins with the expression of *Sry* (Sex-determining region on Y), which suppresses ovarian promoting genes and turns on *Sox9* (Sry-box 9) as part of the testis-determining cascade leading to the activation of *doublesex*/*MAB-3* related transcription factor 1 (*Dmrt1*) and differentiation of Sertoli cells (Veitia 2010). In females, a forkhead box transcription factor (*FoxL2*), β-*catenin* and *Wnt4* are expressed to promote and maintain ovarian development while suppressing *Sox9*. *Dmrt1* contains a DNA-binding motif (DM), and DM domain containing genes and their role in testis differentiation are deeply conserved in diverse organisms from invertebrates to mammals (Matson and Zarkower 2012). Although DM domain genes are deeply conserved in metazoans, up-stream sex regulators such as *Sry*, *Sox*, and *FoxL2* are thought to be recent inventions in vertebrates or placental mammals (Gamble and Zarkower 2012; Matson and Zarkower 2012). In *Caenorhabditis elegans*, an X chromosome to autosome (X:A) ratio of 0.5 triggers expression of a cascade genes that act on three DM domain genes to initiate male development (Gamble and Zarkower 2012). In *Drosophila*, sex is also determined by the X:A ratio through alternative splicing of *doublesex* (*dsx*), a DM domain gene, into *dsx^F^* and *dsx^M^* to initiate female and male differentiation, respectively (Hempel and Oliver 2007; Rideout *et al.* 2010; Robinett *et al.* 2010).

Mollusca is a major branch of lophotrochozoan where diverse modes of sexual reproduction are observed. In bivalve molluscs, there are dioecious and hermaphroditic species, as well as species capable of sex change, and sex determination can be genetic, environmental, or both (Coe 1943; Guo and Allen 1994; Chavez-Villalba *et al.* 2011). Oysters in particular have a complex and dynamic sex determination system. Certain species, including many within the genus *Ostrea*, are functional hermaphrodites, whereas others such as *Crassostrea* species may exhibit protandric dioecy, hermaphroditism and sex change within the same species (Coe 1943). In the Pacific oyster *Crassostrea gigas*, most individuals mature as males first and may subsequently change to females, and females may change to males as well (Amemiya 1929). Warm and nutritious conditions may favor the development of females (Quayle 1988). Genetic determinants of sex in oysters also have been demonstrated through analysis of family sex ratios, and genetic models have been proposed without any knowledge of molecular mechanisms of sex determination (Haley 1979; Guo *et al.* 1998; Hedrick and Hedgecock 2010).

Studies on sex determination genes and pathways in bivalves and other molluscs are few and limited. Several genes homologous to sex-determining pathway genes in model species have been identified in bivalve molluscs, although their expression profile is often inconsistent with expected roles in sex determination. DM domain genes have been identified in *C. gigas*, scallop *Chlamys farreri*, and *Chlamys nobilis*, but they are not specifically expressed in testis (Naimi *et al.* 2009a; Feng *et al.* 2010; Shi *et al.* 2014). Homologs of *Dmrt* did show high or specific expression in the pearl oyster *Pinctada martensii*, the blacklip pearl oyster *Pinctada margaritifera*, and the abalone *Haliotis asinina* (Klinbunga *et al.* 2009; Yu *et al.* 2011; Teaniniuraitemoana *et al.* 2014). *Sox* genes have been reported in *C. farreri* and *C. gigas*, but their expression is not restricted to testis (He *et al.* 2013; Santerre *et al.* 2014). *Cg-FoxL2* and *Cg-β-catenin* showed high but not specific expression in female gonad (Naimi *et al.* 2009b; Santerre *et al.* 2014), although *FoxL2* was later found specifically expressed in female gonad of *C. gigas* and *P. margaritifera* (Dheilly *et al.* 2012; Teaniniuraitemoana *et al.* 2014). Despites the identification of some candidate genes, sex-determining pathways in molluscs remain elusive. Genome-wide studies have been limited due to a lack of reference genomes (Ghiselli *et al.* 2012; Dheilly *et al.* 2012; Teaniniuraitemoana *et al.* 2014).

The availability of the *C. gigas* genome (Zhang *et al.* 2012) provides an opportunity for a comprehensive analysis of sex-determining pathways in this species that has an interesting sex determination system. In this study, we investigated genes related to sex determination by sequencing and analyzing transcriptomes of female and male gonads of *C. gigas*. Our analysis reveals that several key genes of the vertebrate sex-determining pathway are present in *C. gigas* with expression profiles consistent with roles in sex determination, and the *C. gigas* pathway appears more similar to that of vertebrates than to that of worms and flies.

## Methods

To discover genes related to sex-determining pathways, we sequenced transcriptomes of mature gonads from one female (F3) and two male (M2 and M3) Pacific oysters. In addition to the three transcriptomes obtained in this study, we also included gonadal transcriptomes of two females (F1 and F2) and one male (M1) that were generated in our previous study (Zhang *et al.* 2012). Thus, a total of three female and three male gonadal transcriptomes were included in this analysis. To establish expression profile and infer function of selected genes, we consulted seven somatic transcriptomes from six organs (gill, outer and pallial mantle, adductor muscle, digestive gland, labial palp, and hemocytes) and transcriptomes from 38 developmental stages that were obtained in our previous study (Zhang *et al.* 2012).

All animals used for gonadal transcriptome sequencing in this and the previous study were mature 2-yr-old oysters obtained from Laodong Aquaculture Breeding Company, Qingdao, China. Sex of oysters was determined by observing the presence of eggs or sperm under microscope. Gonadal tissues were dissected, frozen in liquid nitrogen and stored at −80° before RNA extraction. RNA extraction, cDNA synthesis, library construction, and transcriptome sequencing were performed in the same way as in our previous study (Zhang *et al.* 2012). In brief, total RNA was extracted using TRIzol reagents and protocol (Invitrogen). Poly-A RNA was isolated with oligo-dT-coupled beads and sheared for first strand cDNA synthesis with random hexamers and Superscript II reverse transcriptase (Invitrogen). The second strand was synthesized with *Escherichia coli* DNA PolI (Invitrogen). Double-stranded cDNA was purified with the Qiaquick PCR purification kit (QIAGEN, Gaithersburg, MD). After end repair and addition of a 3′ dA overhang, the cDNA was ligated to Illumina adapters and size selected to about 200 bp by gel purification. The selected fragments were amplified for 15 polymerase chain reaction cycles and sequenced for 49 bp single-end reads using Illumina sequencing platform. After cleaning, RNA-seq reads were mapped to the oyster genome with Tophat (Trapnell *et al.* 2009). Expression level for each gene was measured with reads per kilobase per million (RPKM). Only genes with a RPKM value larger than 3 in at least 1 of the 13 transcriptomes (6 gonad and 7 somatic) were used for further analysis.

Ovary-specific genes were identified by comparing expression in three female gonadal transcriptomes with that in seven somatic and three testis transcriptomes and vice versa for testis-specific genes. To qualify as ovary or testis-specific genes, genes should satisfy the following criteria: 1) All three ovary or testis samples had greater RPKM values than any of the other 10 samples; 2) The RPKM mean of the three ovary or testis samples should be at least fivefold of that in other 10 samples. For a general characterization of ovary- and testis-specific genes, Gene Ontology (GO) term enrichment analysis was conducted with the Fisher’s exact test in classic algorithm of topGO (Alexa and Rahnenfuhrer 2010). KEGG enrichment was conducted with the algorithm implemented in GOstats (Falcon and Gentleman 2007). The Benjamini-Hochberg false discovery rate control was used to adjust the *P* value (Benjamini and Hochberg 1995).

Genes belonging to sex-determining pathways in model species (see the *Results*) were collected from the literature and searched against the oyster genome and our transcriptome dataset. Homologs were identified and their expression profiles in ovary, testis, somatic tissues, and at various developmental stages were extracted to infer function in *C. gigas*. Although the published oyster genome provided annotation for most genes, genes of interest were manually checked with ESTs and RNA-seq reads for possible assembly errors and characterized by searching against databases, including InterPro (Hunter *et al.* 2009), GO (Ashburner *et al.* 2000), SWISS-PROT (Magrane and Consortium 2011), TrEMBL (Magrane and Consortium 2011), and KEGG (Kanehisa and Goto 2000). Domain structures of selected genes were determined using SMART (http://smart.embl-heidelberg.de/). To confirm homology of selected genes, conserved domain sequences were identified, aligned, and compared. Unrooted maximum likelihood phylogenetic trees of protein sequences were constructed with RAxML (Stamatakis 2014), with the model PROTGAMMAJTT and bootstrap for 1000 replications. Genes in the oyster genome are coded by a prefix of “CGI_100” plus a five-digit unique gene number and for brevity especially in figures, the prefix of “CGI_100” was replaced with “Cg.”

## Results and Discussion

### Ovary- and testis-specific genes

Comparative analyses of RNA-seq data between gonad and somatic organs identified 621 genes that were specifically expressed in female gonad, and 552 specifically expressed in male gonad (Supporting Information, File S1). The finding of more ovary-specific than testis-specific genes is in agreement with the finding of 1570 ovary-enriched and 1370 testis-enriched genes in zebrafish (Santos *et al.* 2007), but differs from other studies where more genes are found in testis (Rinn *et al.* 2004; Small *et al.* 2009). Analyses of these ovary- and testis-specific genes reveal some similarities and interesting differences between two sexes. Genes related to meiosis are overrepresented in both ovary- and testis-specific genes. Among ovary-specific genes, GO terms related to DNA replication, nucleic acid metabolism, DNA repair, chromosome organization, cell cycle, gene expression regulation, DNA recombination, and telomere maintenance are significantly enriched (Table 1). Among testis-specific genes, GO terms related to protein phosphorylation and dephosphorylation, protein metabolism, and sex differentiation are significantly enriched. KEGG enrichment analysis shows similar patterns that pathways related to DNA replication, DNA repair, nucleotide metabolism, recombination, oocyte maturation, cell cycle, and proteolysis are enriched in ovary-specific genes, whereas KEGG pathways related to protein digestion and absorption, protein interaction, flagellar assembly, proteolysis, and focal adhesion are enriched in testis-specific genes (Table 1). These findings suggest that ovary-specific genes are more likely involved in DNA replication, DNA metabolism, DNA repair, DNA organization, and DNA transcription, whereas testis-specific genes are primarily involved in protein phosphorylation, protein interactions and protein metabolism.

**Table 1:** GO terms and KEGG pathways enriched in ovary- and testis-specific genes of *C. gigas*

ID Code	Term	Genes	Expected	*P* value
GO: ovary				
GO:0006260	DNA replication	27	1.7	3.90E-26
GO:0090304	Nucleic acid metabolic process	76	22.7	1.40E-23
GO:0006281	DNA repair	12	2.2	1.80E-06
GO:0008152	Metabolic process	137	108.0	6.60E-06
GO:0006996	Organelle organization	16	4.6	1.30E-05
GO:0000278	Mitotic cell cycle	6	0.6	2.40E-05
GO:0051276	Chromosome organization	10	2.2	5.40E-05
GO:0071103	DNA conformation change	6	1.5	0.00369
GO:0007017	Microtubule-based cellular movement	8	2.7	0.00527
GO:0016070	RNA metabolic process	28	17.1	0.00621
GO:0043687	Posttranslational protein modification	4	1.0	0.01488
GO:0006950	Response to stress	15	8.1	0.01497
GO:0010468	Regulation of gene expression	18	11.4	0.03546
GO:0006310	DNA recombination	2	0.3	0.04507
GO:0000723	Telomere maintenance	1	0.1	0.04857
GO: testis				
GO:0006468	Protein phosphorylation	15	4.6	4.50E-05
GO:0055085	Transmembrane transport	14	6.5	0.00517
GO:0019538	Protein metabolic process	30	19.2	0.00542
GO:0044765	Single-organism transport	20	11.8	0.01116
GO:0016999	Antibiotic metabolic process	1	0.0	0.02340
GO:0006470	Protein dephosphorylation	5	1.8	0.03155
GO:0007548	Sex differentiation	1	0.0	0.03490
GO:0050953	Sensory perception of light stimulus	1	0.1	0.04627
KEGG: ovary				
03030	DNA replication	21	1.5	7.04E-18
04110	Cell cycle	29	3.6	7.04E-18
04113	Meiosis, yeast	19	1.9	8.06E-14
03420	Nucleotide excision repair	15	1.9	1.49E-09
00240	Pyrimidine metabolism	18	3.3	1.11E-08
03440	Homologous recombination	12	1.4	2.41E-08
00230	Purine metabolism	22	6.4	6.67E-07
04914	Progesterone-mediated oocyte maturation	12	1.9	6.67E-07
03430	Mismatch repair	8	1.2	3.98E-05
00785	Lipoic acid metabolism	4	0.3	0.00038
04115	P53 signaling pathway	8	1.9	0.00065
04114	Oocyte meiosis	12	3.9	0.00067
00670	One carbon pool by folate	3	0.6	0.01936
04120	Ubiquitin mediated proteolysis	12	6.4	0.02939
00624	Polycyclic aromatic hydrocarbon degradation	3	0.7	0.03771
KEGG: testis				
04113	Meiosis, yeast	5	0.9	0.01878
04962	Vasopressin-regulated water reabsorption	4	0.7	0.02478
04974	Protein digestion and absorption	6	2.1	0.03946
04512	ECM-receptor interaction	9	4.0	0.03946
02040	Flagellar assembly	1	0.0	0.03946
00230	Purine metabolism	7	3.1	0.03946
04120	Ubiquitin mediated proteolysis	7	3.1	0.03946
04140	Regulation of autophagy	2	0.3	0.03946
04510	Focal adhesion	12	6.8	0.03946

GO, Gene Ontology; ECM, extracellular matrix; KEGG, Kyoto Encyclopedia of Genes and Genomes.

Protein phosphorylation is important for sperm capacitation in mammals (Visconti and Kopf 1998). In mammalian testis, abnormal or damaged spermatozoa are ubiquitinated for proteolytic destruction (Sutovsky *et al.* 2002; Purdy 2008). The fact that ubiquitin mediated proteolysis is enriched among testis-specific genes suggests ubiquitination may play a role in maintaining sperm quality in oysters also. GO terms related to cellular component movement, RNA metabolism, and response to stress are enriched in ovary-specific genes, suggesting these pathways are also important to ovary biology. Overall, it seems that ovary-specific genes are enriched for more diverse pathways than testis-specific genes (Table 1), which may indicate that the production of large and yolk-containing oocytes involves more metabolic pathways than the production of sperm.

### Sex-determining pathway genes

Clearly, not all ovary- and testis-specific genes are involved in sex-determining pathways. To identify genes related to sex-determining pathways, we searched the oyster genome for such genes previously identified in model organisms and examined their expression profiles in gonadal transcriptomes of *C. gigas*. Of the 24 genes examined, homologs were found for nine genes or gene families (Table 2). Among these nine genes, only three genes (*CgDsx*, *SoxH* or *Sry*-like and *FoxL2*) showed sex-specific expression as expected for sex-determining genes. The other six genes (*Fem*, *Gata4*, *Wnt4*, *beta-catenin*, *Run*, *Sox9*) did not show sex-specific expression in our samples (Table 2), suggesting that they may not be involved in sex determination or maintenance in mature gonads. We cannot rule out the possibility that they may have sex-specific expression at an earlier stage.

**Table 2:** Presence and sex-specific expression of key sex-determining pathway genes from *Caenorhabditis elegans*, *Drosophila melanogaster* and *Mus musculus*

Name of genes	*Caenorhabditis elegans*	*Drosophila melanogaster*	*Mus musculus*	*Crassostrea gigas*
*Xol-1*	+/+	−/−	−/−	−/−
*Sdc*	+/+	+/−	+/−	−/−
*Her*	+/+	+/−	−/−	−/−
*Tra*	+/+	+/+	+/−	−/−
*Fem*	+/+	+/−	+/−	+/−
*Fog*	+/+	+/−	+/+	−/−
*Fru*	−/−	+/+	−/−	−/−
*Sis*	−/−	+/+	−/−	−/−
*Run*	+/−	+/+	+/−	+/−
*Sxl*	−/−	+/+	−/−	−/−
*Doa*	−/−	+/+	−/−	−/−
*Gata4*	−/−	−/−	+/+	+/−
*Wt1*	−/−	−/−	+/+	−/−
*M33*	−/−	−/−	+/+	−/−
*Sf1*	−/−	−/−	+/+	−/−
*Mis*	−/−	−/−	+/+	−/−
*Dax1*	−/−	−/−	+/+	−/−
*Sry/Sox30/SoxH*	−/−	−/−	+/+	+/+
*Sox9/SoxE/sox100B*	−/−	+/−	+/+	+/−
*MAB-3/dsx/Dmrt1*	+/+	+/+	+/+	+/+
*FoxL2*	−/−	−/−	+/+	+/+
*Rspo1*	−/−	−/−	+/+	−/−
*Wnt4*	−/−	+/−	+/+	+/−
*Beta-catenin*	−/−	+/−	+/+	+/−

The first + sign indicates presence and the second + sign indicates sex-specific expression or confirmed role in sex-determining pathways.

#### Doublesex and MAB-3 related transcription factor 1 (Dmrt1):

**Figure 1:**
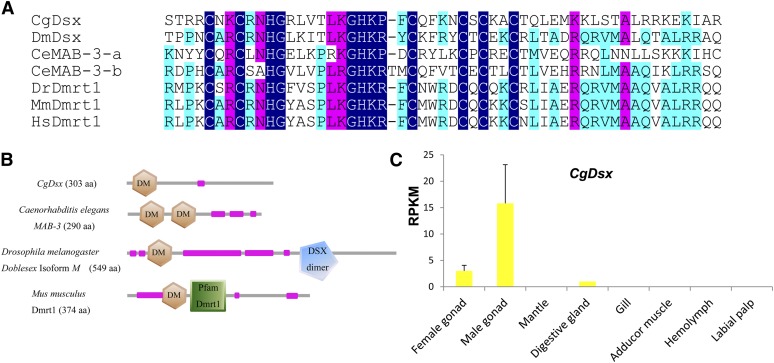
A DM domain gene in *C. gigas* (*CgDsx*) and its male-specific expression profile. (A) Alignment of CgDsx and other DM domain containing proteins involved in sex determination from model species. (B) Domain structure of CgDsx and selected DM domain genes from model species (purple designates low complexity regions). (C) Expression profile of *CgDsx* in different adult organs with standard deviation as error bars (n = 3). Species names are abbreviated as Cg for *Crassostrea gigas*, Dm for *Drosophila melanogaster*, Ce for *Caenorhabditis elegans*, Dr for *Danio rerio*, Mm for *Mus musculus*, and Hs for *Homo sapiens*. Accession numbers: CgDsx KJ489413; DmDsx NP_731197.1, CeMAB-3-a and CeMAB-3-b CAB16489.1, DrDmrt1 AAQ04555.1, MmDmrt1 AAF12826.1, and HsDmrt1 AAD40474.1.

Dmrt1 is a transcription factor that contains a characteristic zinc finger DM domain and plays deeply conserved roles in sex determination and differentiation (Kopp 2012). Members of the family include the *doublesex* (*dsx*) gene in fruit fly, *MAB-3* in *C. elegans* and the *Dmrt1* in vertebrates, all of which promote male-specific development. In *Drosophila melanogaster*, *dsx* is alternatively spliced to produce male- and female-specific isoforms in male and female gonads, respectively (Burtis and Baker 1989). *MAB-3* in *C. elegans* and *Dmrt1* in vertebrates are exclusively expressed in testis and promote male-specific development (Yi and Zarkower 1999; Smith *et al.* 2009; Kopp 2012). The oyster genome encodes three DM domain containing genes: *Cg19568*, *Cg01830*, and *Cg15952*, compared with 11 in nematodes, 4 in flies, and 7 in human (Matson and Zarkower 2012). We named one of the three genes, *Cg19568*, as *CgDsx* (GenBank accession No. KJ489413) after manual correction to remove three misassembled exons. It contains a DM domain showing closest homology (45%, E-value = 9e-13) to *Dsx* isoform A found in *D. melanogaster* (Figure 1A). *CgDsx* has three exons, but it shows no sex-specific alternative splicing as described in *D. melanogaster*. Similar to *MAB-3* from *C. elegans*, *CgDsx* does not contain any other recognizable c-terminal domains that are found in *Dsx* of *Drosophila* or in vertebrate *Dmrt1* (Figure 1B).

*CgDsx* is exclusively expressed in gonads with virtually no expression in somatic organs (0.2 RPKM). Its expression in testis (15.8 RPKM) is 5.3-fold greater than that in ovary (3 RPKM) (Figure 1C). The high expression in testis supports a possible role for *CgDsx* in determining or promoting male-specific development. The finding of low levels of *CgDsx* transcripts in female gonad may indicate that dormant male germline cells exist in female gonad, permitting sex change in coming seasons. Oyster gonads are known to contain both male and female germline cells possibly as a prerequisite for sex change (Cole 1941; Guo *et al.* 1998).

The other two DM domain genes (*Cg15952* and *Cg01830*) both have a DmrtA domain c-terminal to the DM domain and show the highest homology to *DmrtA2* (aka *Dmrt5*) from many species (identity 38–83%, E-value < e-40). They are expressed in all organs and appeared to be unrelated to sex determination or differentiation. *Cg15952* has been previously identified as *Cg-DM1* and shown to be related to gonad development by Naimi *et al.* (2009a). In our data, however, *Cg-DM1* is primarily expressed in gill, labial palp, and mantle (61.5 RPKM) rather than in female (5.3 RPKM) or male (19.6 RPKM) gonads. Our results suggest that *Cg-DM1* may have broad functions in organ development and may not be involved in sex determination in *C. gigas*. *Cg01830* has low expression in gonads (2.9 RPKM), and its greatest expression is at the umbo larval stages (35.6 RPKM) and in adult labial palp (15.1 RPKM), suggesting it is also unrelated to sex determination. Our analysis indicates that this novel *CgDsx* is a DM domain gene and a close relative of *Dsx* that may be involved in promoting male development in *C. gigas*. This finding along with reports of testis-specific Dmrt-like genes in other molluscs (Klinbunga *et al.* 2009; Yu *et al.* 2011; Teaniniuraitemoana *et al.* 2014) supports the idea that the sex-determining role of DM domain genes is deeply conserved in evolution (Matson and Zarkower 2012).

#### Sox genes:

*Sox* (Sry-related HMG box) proteins are a family of transcription factors that possess a DNA binding HMG-box (high mobility group) domain. *Sox* genes are highly conserved in animal kingdom and play key roles in determining cell fate in development and differentiation (Lefebvre *et al.* 2007). Some members of the *Sox* family function in sex determination and differentiation, including the founding member *Sry* (sex-determining region on the Y-chromosome) and *Sox9* from mammals (Kent *et al.* 1996; Koopman 2001; Kashimada and Koopman 2010). Expression of *Sry* activates *Sox9* and the male determining pathway leading to *Dmrt1* (Koopman 2005). *Sox9* inhibits ovary development through the induction of anti-Müllerian hormone in Sertoli cells and promotes male sex-development through the activation of glia-activating factor 9 (Schepers *et al.* 2003). It is specifically expressed in testis germ cells in humans (Su *et al.* 2004).

**Figure 2:**
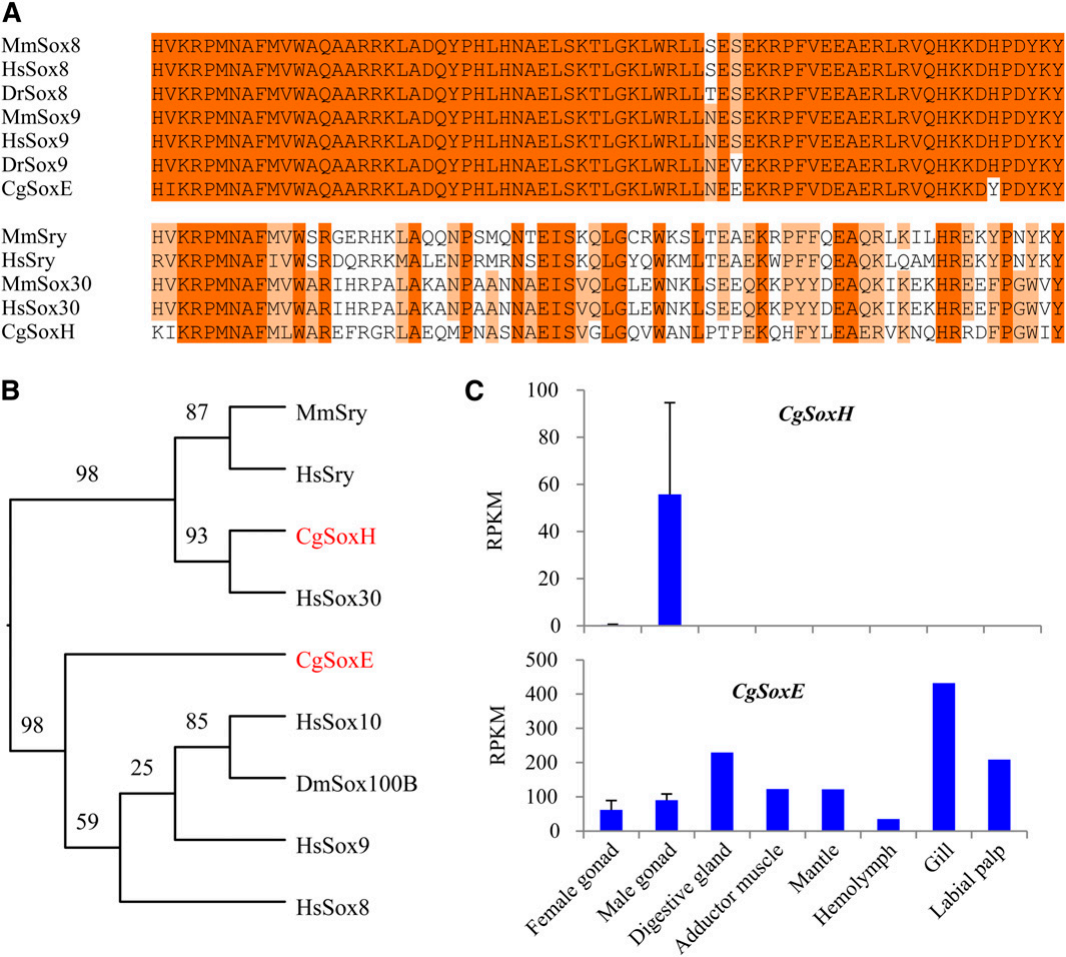
*SoxE* and *SoxH* genes identified in *C. gigas* and their expression profile. (A) Alignment of HMG domains of *CgSoxE*, *CgSoxH*, and homologs from selected vertebrates. (B) Phylogenetic tree of protein sequences of *CgSoxE*, *CgSoxH*, and selected genes. (C) Expression profile of *CgSoxE* and *CgSoxH* in adult organs of *C. gigas* with standard deviation as error bars (n = 3). Species names are abbreviated as Cg for *Crassostrea gigas*, Dm for *Drosophila melanogaster*, Dr for *Danio rerio*, Mm for *Mus musculus*, and Hs for *Homo sapiens*. Numbers in the tree represent bootstrap values. Accession numbers: CgSoxE EKC31659.1, CgSoxH EKC38002.1, DmSox100B AAF57112.2, MmSry AAI11529.1, HsSry AFG33955.1, HsSox8 AAH31797.1, HsSox9 CAA86598.1, HsSox10 CAG30470.1, and HsSox30 BAA37146.1.

The *C. gigas* genome encodes 32 proteins containing the HMG domain, and 10 of them can be classified as *Sox* genes (Table 3). One of the oyster *Sox* genes (*Cg22931*) recently has been identified as a member of the *SoxE* family (*Cg-SoxE*) that includes the sex-determining *Sox9* from mammals (Santerre *et al.* 2014). Its HMG domain shares high similarity (86.1%) to that of *Sox8* and *Sox9* in vertebrates (Figure 2, A and B). However, *Cg-SoxE* is probably not involved in sex determination. In Santerre *et al.*’s (2014) study, *Cg-SoxE* was expressed in both male and female gonads, higher at undifferentiated than mature stages. In our transcriptome data, it was mostly expressed in somatic organs (averaging 197 RPKM) and at gastrula stage (194 RPKM); its expression was lower in male (90 RPKM) and female (61 RPKM) gonads (Figure 2C). These results suggest that the primary function of *Cg-SoxE* may not be related to sex determination or differentiation in *C. gigas*.

**Table 3:** Sox genes in *C. gigas* genome and their expression in gonads

Gene ID	Homolog	E-value	Gonad-specific
*Cg27966*	*Sox4*, *Columba livia*, EMC90062.1	5e-49	No
*Cg10085*	*Sox2*, *Pinctada fucata*, AGS18764.1	2e-124	No
*Cg03991*	*Sox7*, *Homo sapiens*, NP_113627.1	7e-32	No
*Cg05643*	*SoxB2*, *Azumapecten farreri*, AGY14563.1	2e-109	No
*Cg22931*	*Sox9* (*Cg-SoxE*), *Pinctada fucata*, AGI96396.1	8e-161	No
*Cg21811*	*SoxC*, *Platynereis dumerilii*, CAY12635.1	4e-91	No
*Cg05340*	*Sox2*,*Caenorhabditis remanei*, XP_003118339.1	6e-08	No
*Cg06950**^a^*	*Sox30* (group H), *Homo sapiens*, NP_848511.1	5.8e-14	Testis
*Cg03963*	*Sox1* (groupB1), *Xenopus laevis*, NP_001079136.2	9e-79	No
*Cg27723*	*Sox7*-like, *Saccoglossus kowalevskii*, NP_001158464.1	3e-85	No

a *CgSoxH* or *Sry*-like, specifically expressed in testis.

Among all *Sox* genes that we identified in *C. gigas*, only one showed testis-specific expression in our transcriptome data. This novel *Sox* gene *Cg06950*, classified as *CgSoxH*, is closely related to *Sox30*, a member of *SoxH*. Within family identity in vertebrate *Sox* gene families usually ranges between 70 and 95% (Lefebvre *et al.* 2007), and the classification of *CgSoxH* is tentative and needs to be confirmed in future studies. Nevertheless, phylogenetic analysis shows *CgSoxH* is a close relative of *Sox30* and *Sry* of vertebrates (Figure 2B). *CgSoxH* encodes a protein of 1283 aa divided into eight exons, whereas *Sox30* in mammals has five exons alternatively spliced producing two polypeptides of 753 and 501 amino acid residues (Osaki *et al.* 1999). Its HMG domain shares a 54% identity (e-value = 1.4e−15) with *Sox30* from human as well as a 49% identity (e-value = 2e−21) to *Sry* from *Mus musculus domesticus* (Figure 2A). Phylogenetic analysis also shows that *CgSoxH* is clustered with *Sox30* and then with *Sry* (Figure 2B). At four positions, *CgSoxH* shared identical amino acids with *Sry* instead of *Sox30* so it may be considered as an Sry-like gene. It is possible that *CgSoxH* may be closely related to the ancestral gene before the divergence of *Sry* and *Sox30*.

In our data, *CgSoxH* is exclusively expressed in testis (Figure 2C). Its expression in testis is 55.7 RPKM compared with <1 RPKM in all other transcriptomes, including ovary, all somatic organs, and at all developmental stages. In mammals, *Sry* is the primary male-determining gene, and *Sox30* is exclusively expressed in normal testis, but not in maturing germ cell-deficient testis, suggesting a role in differentiation of male germ cells (Osaki *et al.* 1999). The homology between *CgSoxH* and male-determining *Sox30* and *Sry* and the fact that *CgSoxH* is exclusively expressed in testes suggest that *CgSoxH* may play a key role in determining or promoting male sex development in oysters. As far as we can determine, *CgSoxH* is the first *Sry*-like gene with possible roles in male-determination identified in a mollusc. A member of *SoxE* family (*Sox100B*) has been found in *D. melanogaster* showing male-specific expression, although it is clearly not a homolog of *Sry* (Figure 2, A and B). *SoxE* genes have been identified in molluscs but without male-specific expression or roles in sex determination (He *et al.* 2013; Santerre *et al.* 2014, this study). It has been suggested that *Sry* and *Sox9* assumed their roles in sex determination in vertebrates (Matson and Zarkower 2012). The finding of an Sry-like gene in *C. gigas* with testis-specific expression suggests that *Sry* and its role in sex determination may not be inventions specific to vertebrates but deeply rooted in evolution. It is possible that an Sry-like gene existed in the common ancestor of bilaterians and had a role in sex determination already. The gene may have been lost in some lineages (such as worms and flies) but conserved in others (molluscs and vertebrates).

All other oyster *Sox* genes are not specifically expressed in gonads. Many of them are highly expressed at certain embryonic stages, suggesting that they may play roles in determining cell fate during early development.

#### Fox genes:

Fox (forkhead-box) proteins are a family of transcription factors with a characteristic DNA-binding forkhead domain. They regulate gene expression and play roles in diverse biological processes including development, differentiation, metabolism and immunity. One member of the Fox gene family, *FoxL2*, is a key gene involved in ovarian determination in vertebrates (Uhlenhaut and Treier 2006). In mammals, *FoxL2* is expressed in the ovary and promotes ovarian development while suppressing the key male promoting *Sox9* gene (Crisponi *et al.* 2001; Ottolenghi *et al.* 2007; Uhlenhaut *et al.* 2009).

**Figure 3:**
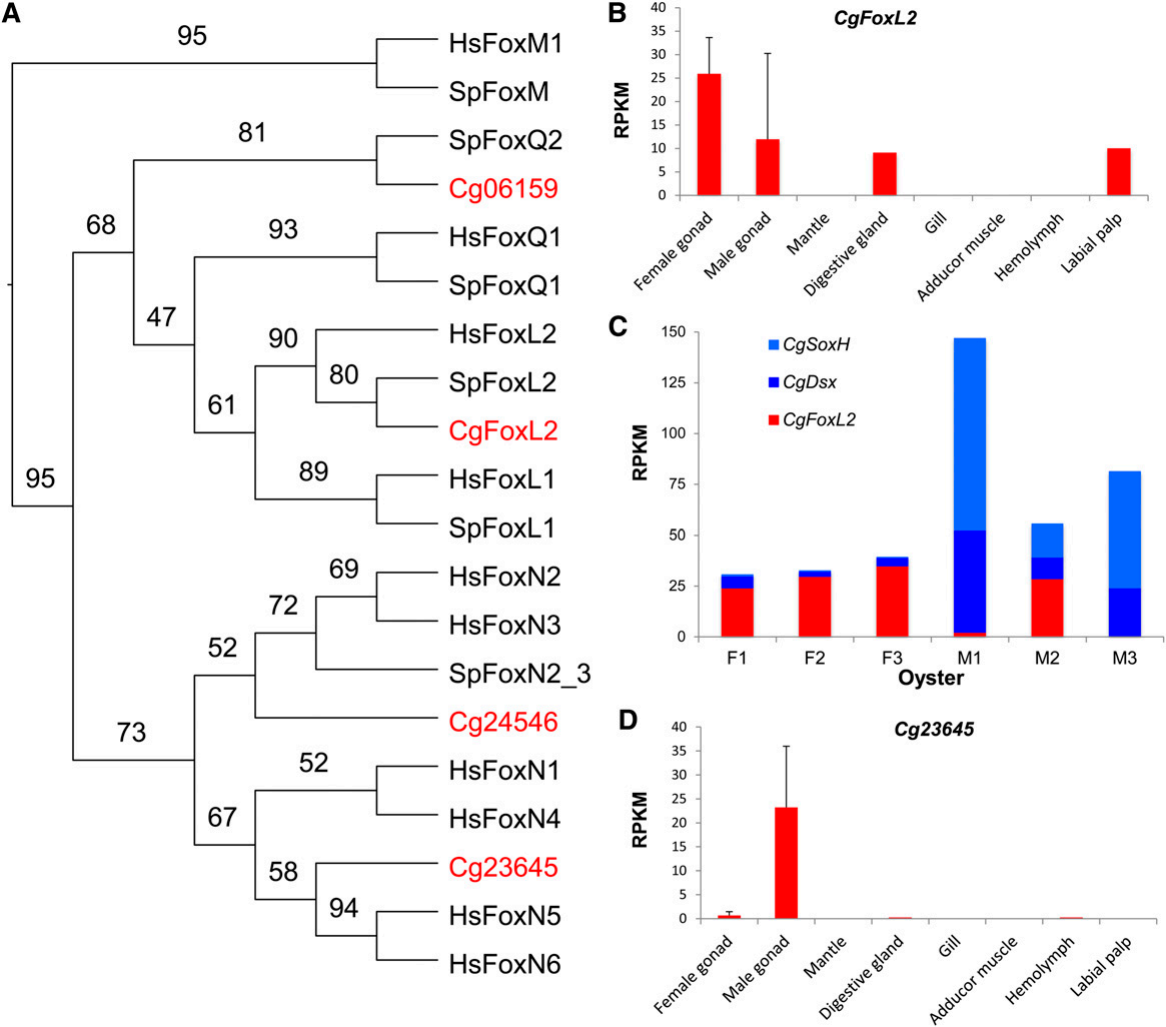
Analysis of selected Fox genes identified in *C. gigas*. (A) Phylogenetic tree of forkhead domains from selected Fox genes from *C. gigas* and reference species. (B) Expression profile of *CgFoxL2* showing high expression in female gonad. (C) Expression of *CgFoxL2*, *CgSoxH*, and *CgDsx* in three females and three males showing possible interaction among the three genes in M2. (D) Male-specific expression of a novel Fox gene *Cg23645*. Error bars represent standard deviation (n = 3). Species names are abbreviated as Cg for *Crassostrea gigas*, Hs for *Homo sapiens* and Sp for *Strongylocentrotus purpuratus*. Numbers in the tree are bootstrap values. Accession numbers: CgFoxL2 ACN80999.1, Cg06159 EKC20378.1, Cg24546 EKC30312.1, Cg23645 EKC35023.1, SpFoxL1 ABB89488.1, SpFoxL2 ABB89483.1, SpFoxM, ABB89490.1, SpFoxN2/3 ABB89482.1, SpFoxQ1 ABB89489.1, SpFoxQ2 ABB89473.1, SpFoxY AF517552, HsFoxL1 AAG40312.1, HsFoxL2 AAY21823.1, HsFoxM1 NP_973731.1, HsFoxN1 NP_003584.2, HsFoxN2 AAH63305.1, HsFoxN3 AAH07506.1, HsFoxN4 AAI46826.1, HsFoxN5 AAH38969.2, HsFoxN6 AAH12934.1, and HsFoxQ1 AAH53850.1.

The *C. gigas* genome encodes 26 Fox genes (Table 4), compared with more than 40 found in human (Katoh and Katoh 2004a). All 26 Fox genes showed evidence of expression in our transcriptome data with two (*Cg06159* and *Cg24546*) specifically expressed in ovary. We initially suspected that these two ovary-specific Fox genes might function like *FoxL2* and determine ovarian development in *C. gigas*, however their expression profiles suggested otherwise. These two Fox genes had their greatest expression in oocytes, which progressively declined during embryonic development, to undetectable levels by late D-stage (*Cg24546*) or juvenile stage (*Cg06159*). They likely play important roles in embryogenesis, but seem unrelated to sex-determining pathways. *Cg06159* is a homolog of *FoxQ2* (Figure 3A). In sea urchin, *FoxQ2* is progressively restricted to the animal plate during cleavage stage and provides the linkage of the primary animal-vegetal and secondary oral-aboral axes (Yaguchi *et al.* 2008). Hydrozoan has two *FoxQ* genes: *FoxQ2a* is expressed in early embryos and maintained through larval stages, while *FoxQ2b* is not expressed in embryos or larvae or polyp, but specially expressed in the gonad of medusa (Chevalier *et al.* 2006). *Cg24546* is closely related to *FoxN2* (Figure 3A), which in murine is involved in differentiation of multiple tissues during embryogenesis (Tribioli *et al.* 2002). In sea urchin, *FoxN2/3* is a key gene involved in the formation of the larval skeleton (Rho and Mcclay 2011).

**Table 4:** Fox genes identified in *C. gigas* genome and their expression in gonads

Gene ID	Homolog	E-value	Gonad specific
Cg25509	Fork head protein, *Patella vulgate*, CAD45552.1	1e-115	No
Cg11405	FoxB1, *Saccoglossus kowalevskii*, NP_001158435.1	8e-78	No
Cg11631	FoxA/B, *Saccoglossus kowalevskii*, NP_001164676.1	1e-71	No
Cg17698	FoxC-like protein, *Saccoglossus kowalevskii*, NP_001158465.1	5e-97	No
Cg11851	FoxD, *Saccoglossus kowalevskii*, NP_001164677.1	3e-69	No
Cg06006	FoxE1, *Saccoglossus kowalevskii*, NP_001158436.1	4e-37	No
Cg08560	FoxF, *Patella vulgate*, CBI70345.1	5e-88	No
Cg28651	FoxG, partial, *Terebratalia transversa*, AEZ03828.1	8e-79	No
Cg21832	FoxJ1, *Saccoglossus kowalevskii*, NP_001158438.1	3e-66	No
Cg19731	FoxJ2/3, *Saccoglossus kowalevskii*, ADB22670.1	6e-105	No
Cg26255	FoxK1, *Branchiostoma floridae*, ACE79146.1	8e-175	No
Cg17701	Fox/forkhead, *Capitella teleta*, ADC35033.1	1e-65	No
Cg11004	*FoxL2*, *Azumapecten farreri*, AFB35647.1	4e-132	No*^a^*
Cg06326	FoxM1, partial, *Columba livia*, EMC82562.1	2e-28	No
Cg14633	FoxN1, *Saccoglossus kowalevskii*, NP_001158439.1	3e-60	No
Cg11126	FoxN2/3, *Branchiostoma floridae*, ACE79140.1	2e-91	No
Cg24546	FoxN2, *Bos mutus*, ELR49084.1	8e-15	Ovary*^b^*
Cg07980	FoxO, *Blattella germanica*, CCF23214.1	1e-96	No
Cg14285	FoxP, *Octopus vulgaris*, ACN38054.1	2e-113	No
Cg06159	FoxQ2-like, *Saccoglossus kowalevskii*, NP_001161546.1	5e-51	Ovary*^b^*
Cg02561	Foxl1-ema, *Chelonia mydas*, EMP25610.1	4e-46	No
Cg03726	FoxQ/D-like protein, *Saccoglossus kowalevskii*, NP_001161545.1	9e-74	No
Cg12628	FoxH1, *Dicentrarchus labrax*, CBN81873.1	2e-27	No
Cg01578	FoxH1, *Oryzias latipes*, NP_001153943.1	8e-07	No
Cg23645	Fox protein, *Glarea lozoyensis*, EPE29336.1	3e-06	Testis

a Highly expressed in ovary, although technically not classified as ovary-specific because of abnormally high expression in one male.

b Greatest expression is in oocytes.

A homolog of *FoxL2* has previously been identified in *C. gigas* (Naimi *et al.* 2009b), which is also identified in the oyster genome assembly (Figure 3A) although the assembled copy (*Cg11004*) incorrectly included an additional 3′ exon. In Naimi *et al.*’s study, *CgFoxL2* is expressed in both female and male gonads, with a significant increase in females earlier during sexual development. In our transcriptome data, *CgFoxL2* is also expressed in both sexes, highly although not specifically expressed in the ovary (due to abnormally high expression in one male) (Figure 3B). Its expression is high in gonads of all three females (24–35 RPKM) and one male (29 RPKM), but low in the other two males (0.3−2.2 RPKM). Without the exceptional male (M2 in Figure 3C), *CgFoxL2* would qualify as an ovary-specific gene (Dheilly *et al.* 2012). Although the number of oysters sampled is small, the large variation in males is consistent with the sex determination model that recognizes two types of males: fake males that change sex and true male that do not (Guo *et al.* 1998). M2 with high *CgFoxL2* expression may be a fake male that has a higher tendency to change sex. Interestingly, the exceptional male (M2) with high *CgFoxL2* expression also had exceptionally low expression of male-promoting *CgDsx* and *CgSoxH* genes (Figure 3C). It is possible that all three genes collectively and through their interactions make M2 prone to sex change. This is largely speculative at this time but can be tested in future studies.

*CgFoxL2* is not expressed in oocytes, early embryos, or somatic organs except at moderate levels in digestive gland (possibly due to contamination by gonad) and labial palp (Figure 3B). A small peak of *CgFoxL2* expression is observed at trochophores stage, which together with ovary-specific expression during sexual development, point to a likely role for *CgFoxL2* in germline or ovarian determination.

It is interesting to note that one Fox gene (*Cg23645*) is specifically expressed in testis (Figure 3D). *Cg23645* or *CgFoxN5* is closely related to *FoxN5*, which in mouse is expressed in embryonic germ cells and zygote (Katoh and Katoh 2004b). *CgFoxN5* is not expressed at embryonic and larval stages, nor in any somatic organs in *C. gigas*. Its highly specific expression in testis is novel and may point to a possible role for this novel Fox gene in testis development in *C. gigas*.

### A working model for sex determination in *C. gigas*

The Pacific oyster has a complex sex-determining system that is characterized by protandry, sex change, and rare but consistent hermaphroditism, and how such a dynamic system is controlled and maintained has been the subject of considerable interest (Coe 1943, Haley 1979, Guo *et al.* 1998, Naimi *et al.* 2009a,b, Hedrick and Hedgecock 2010, Santerre *et al.* 2014). We identified possible sex-determining pathway genes in *C. gigas* based on sequence homology and functions inferred from transcriptome data. The assumption is that if the gene is involved in sex determination in other organisms and it shows sex-specific expression that is consistent with its known function, it may be related to the sex-determining pathway in *C. gigas*. We recognize that sequence homology and expression data can only identify possible candidates that require further experimental verification. Given the state of knowledge about sex determination in molluscs, identification of candidate genes and working models are necessary steps for further analysis.

**Figure 4:**
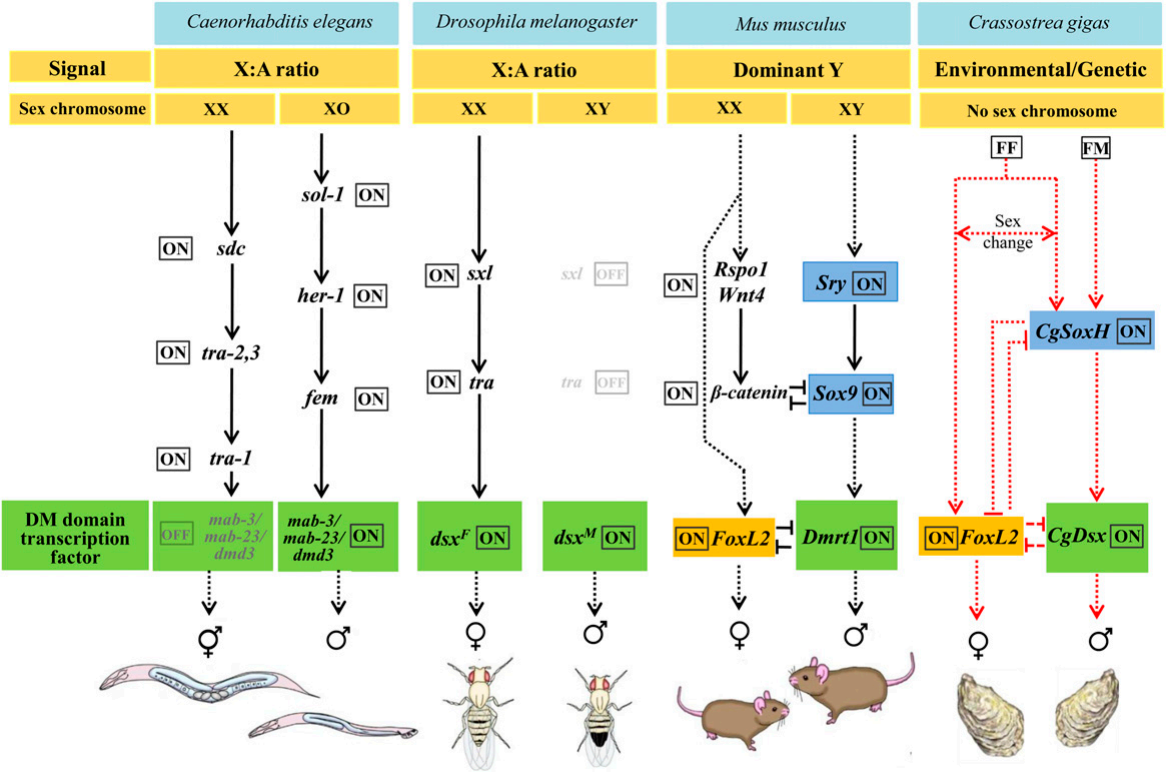
Hypothesized sex-determining pathway in *C. gigas* compared with that in model organisms as summarized by Gamble and Zarkower (2012). For clarity, only selected key sex-specific regulators are shown. Dashed black lines indicate temporal relationships, and dashed red lines indicate hypothetical relationships based on expression data only. [Modified based on Gamble and Zarkower (2012)]. FF genotype permits sex change (Guo *et al.* 1998).

Our analyses indicate that *CgDsx*, *CgSoxH*, and *CgFoxL2* are probably involved in sex determination in *C. gigas*. All three genes or their close relatives are key elements of sex-determining pathways in vertebrates and exhibited sex-specific expressions. Other than the DM domain containing *Dsx* that has been shown to have a deeply conserved role in sex determination in both invertebrates and vertebrates, *Sry* and *FoxL2* are thought to be new recruits to sex-determining pathways in vertebrates or placental mammals (Gamble and Zarkower 2012; Matson and Zarkower 2012). The finding of these key vertebrate sex-determining genes with expected expression profile for sex determination in *C. gigas* is novel and suggests that these vertebrate genes may not be inventions of vertebrates. Their role in sex determination may be deeply rooted in evolution and at minimum conserved in a mollusc, despite rapid evolution of the regulatory pathways that in *C. gigas* may involve both genetic and environmental factors. Except for the deeply conserved DM domain gene *Dsx*, sex-determining or regulating genes in *C. elegans* and *D. melanogaster* are either not found in *C. gigas* or without expression profiles expected for sex-determining pathway genes (Table 2 and Figure 4). Our analysis suggests that sex determination in *C. gigas* may share more similarities with that in vertebrates than with that of worms and flies (ecdyspzoans). Although the three groups of bilaterians, Lophotrochozoa, Ecdysozoa, and Deuterostomia, are well-recognized, their relationship to each other is not clear. Phylogenetic analysis based on whole-genome sequences indicates that although molluscs and annelids (lophotrochozoans) are related to worms and flies within protostomes, their genomes in many aspects are more similar to those of invertebrate deuterostomes (Simakov *et al.* 2013). Also, molluscs share the same telomeric sequence with vertebrates, but not with worms and flies (Zakian 1995, Guo and Allen 1997, Sakai *et al.* 2005). The conservation of genes related to sex determination between the oyster and vertebrates provides additional argument that molluscs may be closer to the common bilaterian ancestor than ecdysozoans.

As a working model, we speculate that *CgSoxH* may play a leading role in the sex-determining pathway of *C. gigas* as it is closely related to the up-stream regulator *Sry* in vertebrates and strictly expressed in testis. It may directly or indirectly activate *CgDsx* (Figure 4), which as a DM domain gene is a master switch for testis development in all metazoans studied so far. DM domain genes have been suspected for roles in male-determination in bivalve molluscs (Yu *et al.* 2011). Both *CgSoxH* and *CgDsx* may interact or inhibit *CgFoxL2*, which is specifically expressed in ovaries with the exception of one male (M2 in Figure 3C and partly supported by Naimi *et al.* 2009b). The abnormally high expression of *CgFoxL2* and low expression of *CgSoxH* and *CgDsx* in M2 provided preliminary evidence for possible interaction among these male- and female-promoting genes (Figure 3C). This finding is preliminary but consistent with the reported interaction among *Sry*, *Sox9*, *Dmrt1*, and *FoxL2* in mammals (Veitia 2010, Matson *et al.* 2011).

The finding of large variation in expression of sex-determining genes in males supports one of the genetic models of sex determination that recognizes two types of males: fake males (FF) that change sexes and true males (FM) that do not (Guo *et al.* 1998). Although the variation could be caused by various factors such as different stages of sexual development, it is possible that M2 is a FF male where low expression of male-promoting *CgSoxH* results in low expression of male-promoting *CgDsx* and high expression of female-promoting *CgFoxL2*, which in turn may promote sex change to female. The number of oysters studied here is limited, and further studies are needed. If the proposed model is correct, it would be interesting to ask how the expression of *CgSoxH* is controlled by cis/trans genetic elements and by environmental factors. The working model and insights provided in this study should stimulate further investigation on sex-determining pathways in molluscs and other invertebrates.

This study identified two novel genes, *CgDsx* and *CgSoxH* (or *Sry*-like), that are likely involved in sex determination in *C. gigas* and provided supporting data for the involvement of *CgFoxL2*. The sex-determining functions of *Sry* and *FoxL2* are thought to have emerged late during the evolution of vertebrates. The finding of such genes in *C. gigas* with sex-specific expression indicates that these vertebrate sex-determining genes may not be inventions of vertebrates as suggested by previous studies. Their role in sex determination may be deeply conserved in evolution, despite rapid evolution of the regulatory pathways that in *C. gigas* may involve both genetic and environmental factors.

## Supplementary Material

Supporting Information
