# Supplementary material for: Genomic Analysis of the Pacific Oyster (Crassostrea gigas) Reveals Possible Conservation of Vertebrate Sex Determination in a Mollusc
Source: G3 (Bethesda). 2014 Sep 11;4(11):2207–17. doi: 10.1534/g3.114.013904 (PMC4232546; doi:10.1534/g3.114.013904)
Supplement: Supporting Information [file supp_4_11_2207__index.html]

Genomic Analysis of the Pacific Oyster (Crassostrea gigas) Reveals Possible Conservation of Vertebrate Sex Determination in a Mollusc — Genomic Analysis of the Pacific Oyster (Crassostrea gigas) Reveals Possible Conservation of Vertebrate Sex Determination in a Mollusc — Supporting Information 

# Genomic Analysis of the Pacific Oyster (*Crassostrea gigas*) Reveals Possible Conservation of Vertebrate Sex Determination in a Mollusc

## Supporting Information for Zhang, Xu, and Guo, 2014

**Files in this Data Supplement:**

- File S1 - Ovary- and Testis specific genes identified in *Crassostrea gigas* and their expression profile in ovary, testis and somatic organs. Raw sequence reads have been submitted to NCBI Sequence Read Archive under accession #SRR1501077. (.xls, 424 KB)
